# Integrating Humanities and Arts Into Gerontology and Geriatrics Curricula

**DOI:** 10.1093/geroni/igab046.263

**Published:** 2021-12-17

**Authors:** Desmond O'Neill, Dana Bradley, James Powers

## Abstract

Humanities, Arts and Cultural Gerontology (HACG) has been an integral element of GSA for over 4 decades, and is included as a notable feature of AGHE guidelines on curricula for gerontology and geriatrics. However, as with many interdisciplinary areas, the degree to which HACG has been successfully inserted into curricula, the extent to which this has involved engagement of faculty in arts and humanities, and the facilitators and barriers of such deeper joint working are unknown. The HACG Advisory Panel and AGHE would like to convene a round-table/symposium at the 2021 Phoenix GSA Meeting to consider the range of experiences of educators of programs in gerontology/geriatrics, from those who can relate success stories in integrating HACG into their curricula, to those who can give insights into challenges and opportunities in attempts to develop such elements in their curricula. Co-convened by Des O'Neill, Chair HACG AP and Dana Burr Bradley AGHE Program Chair, we invite lively discussion which we consider will aid in the development of a road map towards substantive and rewarding initiatives in incorporating scholarship and education in HACG in gerontology and geriatrics educational program

